# Flow dynamics in acute ischemic stroke due to embolic occlusion of a fetal posterior cerebral artery treated with endovascular thrombectomy - report of two cases

**DOI:** 10.1016/j.radcr.2022.01.025

**Published:** 2022-03-24

**Authors:** Karl Matz, Andrei Apetroe, Andreas Chemelli, Cornelia Brunner, Christian Nasel

**Affiliations:** a University of Continuing Education Krems, Department for Clinical Neurosciences and Preventive Medicine, 3500 Krems, Austria; b Landes Klinikum Baden-Mödling, Neurological Departement, 2340 Mödling, Austria; c Landes Klinikum Baden-Mödling, Radiological Departement, Mödling, Austria; d University Clinic Tulln, Landsteiner Private University, Neurological Department, Tulln, Austria; e University Clinic Tulln, Landsteiner Private University, Radiological Departement, Tulln, Austria; f Center for Medical Physics and Biomedical Engineering, Medical University of Vienna, Vienna, Austria

**Keywords:** Fetal circle of Willis, Acute ischemic stroke, Thrombectomy, Stent, Retriever, Cerebrovascular circulation

## Abstract

The fetal variant of the posterior cerebral artery (fPCA) conserves a major blood flow from the anterior to the posterior cerebral circulation via a strong persistent caudal portion of the embryonic internal carotid artery. We present two cases where endovascular treatment in acute ischemic stroke was complicated by this flow diversion. Though direct thrombectomy of the fPCA using a stent retriever was feasible and successful in both cases outcome remained unfavourable due to a continuous redirection of embolic material into the posterior circulation. Knowledge of flow dynamics in a fPCA is important for endovascular treatment in acute ischemic stroke.

## Introduction

Cerebral endovascular thrombectomy (cTE) significantly extended the therapeutical possibilities of treatment of acute ischemic stroke and is recommended in all guidelines for treatment of occlusions of large cerebral vessels, such as the M1 and proximal M2 segments of the middle cerebral artery (MCA), the intracranial internal carotid artery (ICA) and the basilar artery [1]. Several large randomized controlled trials proved the efficacy of cTE even up to 24 hours in patients with a persistent so-called extended ischemic mismatch [2,3].

However, concerning occlusions of the posterior cerebral artery (PCA) data from randomised trials are scarce and, since deficits associated with PCA-infarctions are perceived as less impairing compared to those of MCA-infarctions, an occlusion of the PCA is currently not considered a definite target of the endovascular approach. Still, some case series and multicentre registries suggested efficacy of cTE in PCA occlusions usually reporting a high recanalization success even in the distal P2 or P3 segments [4,5]. In this context, only few is known about cerebral hemodynamics and their impact on the treatment of acute ischemic stroke in case of an occlusion of a persistent caudal portion of the embryonic ICA forming a fetal PCA variant (fPCA) supplying the posterior cerebrovascular territories including the thalamic nuclei. This variant occurs in up to 26% of explored subjects and is supposed to potentially redirect the embolic trajectory from the ICA toward the posterior cerebral circulation [6–8]. Therefore, a deeper understanding of the vascular flow and cerebral perfusion dynamics in case of a fPCA is of interest. Here, we report two cases of embolic occlusion of a fPCA treated with cTE and intravenous thrombolysis (ivTL).

## Methods

The patients’ clinical histories and imaging data from the primary admitting stroke unit (adStU) and the interventional stroke center (intStC) were reviewed retrospectively.

Additional data was received from the endovascular part of the Austrian Stroke Unit registry [8,9]. Quantitative perfusion magnetic resonance imaging (P-MRI) was assessed by calculation of the standardized time-to-peak parameter (stdTTP, jPerfusionTool, C.N.) with interpretation of findings according to the proposed 3- range model [9]. Vessel occlusions depicted in intra-arterial digital subtraction angiographic (iaDSA) images were scored according to the original 5-point thrombolysis-in-cerebral-infarction (TICI) scale [10]. Management of patients and processing of data were handled following ethical principles for the medical research involving human subjects according to the World Medical Association Declaration of Helsinki [11].

### Case 1

**Fig. 1:**
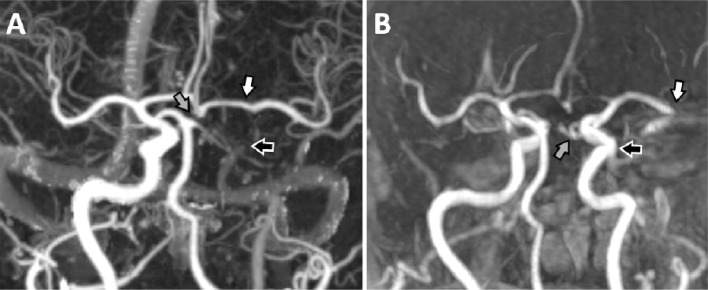
Case 1 - CTA (A) of case 1 performed 3 hours after symptom onset. An occlusion of the left intracranial ICA (black arrow) with preserved filling of the left MCA (white arrow) was visible. Note the missing depiction of the left PCA (grey arrow). MRA (B) performed right before thrombectomy revealed a complete recanalisation of the left ICA (black arrow) and partial filling of the left fPCA (grey arrow). Migration of the initial thrombus to the major central M2-branch of the left MCA (white arrow). CTA, CT-angiography; fPCA, fetal posterior cerebral artery; ICA, internal carotid artery; MCA, middle cerebral artery; MRA, MR angiography.

A 79-year-old female was admitted to the adStU with a left hemispheric stroke syndrome with severe sensorimotor aphasia, right sided gaze palsy and right sided hemiparesis. Symptom onset was 3 hours before and Nationals Institute of Health Stroke Scale (NIHSS) at admission was 14. Initial CT of the brain was unremarkable without early infarct signs. CT-angiography (CTA) suggested an occlusion of the C4-segment of the left ICA, but complete filling of the left MCA was preserved via cross flow from the contralateral side. The left PCA was not visible (Fig. 1A).

**Fig. 2:**
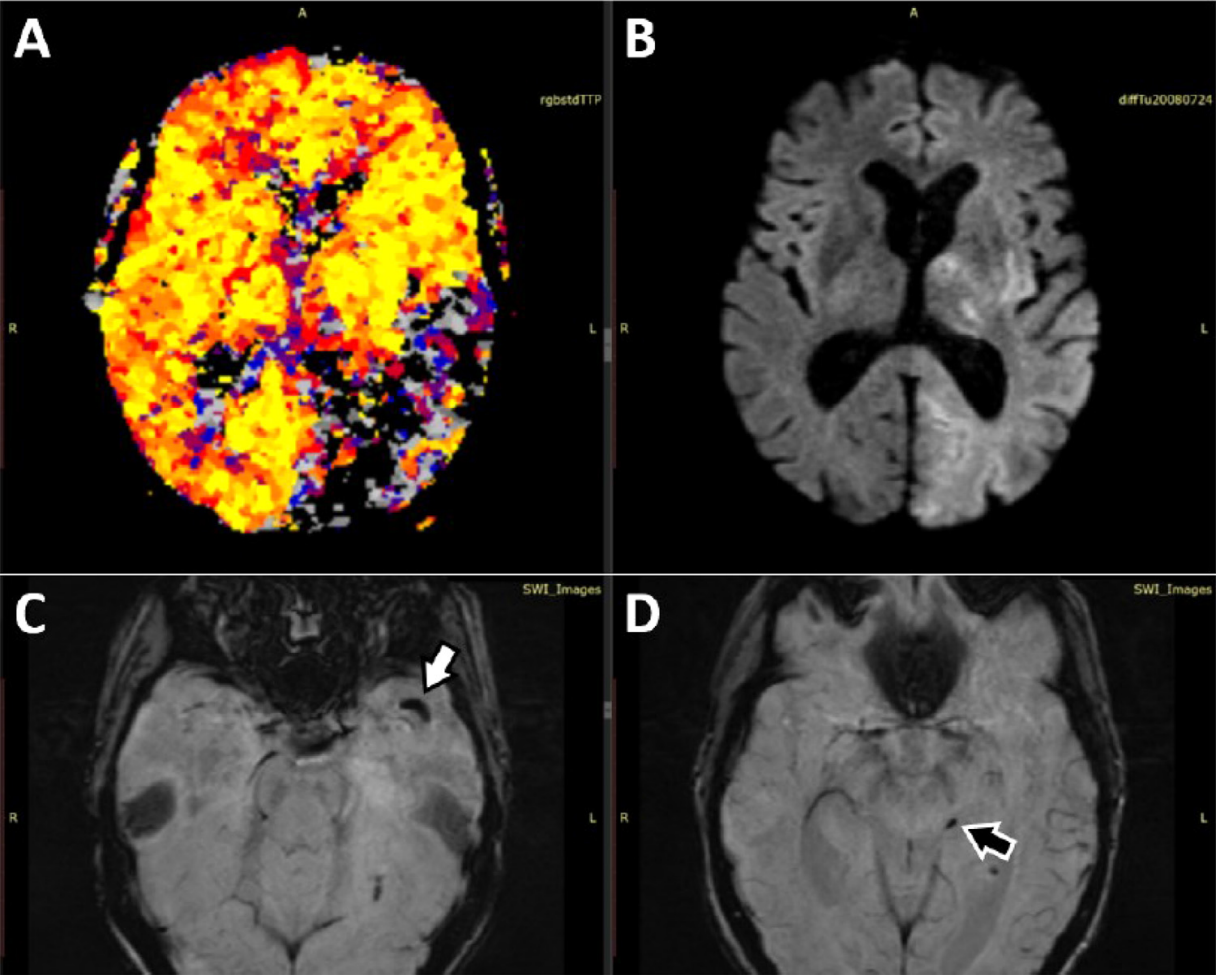
Case 1 - Geometrically matched stdTTP-P-MRI (A) and DWI (B) of case 1 before cTE. After ivTL, migration of the thrombus initially located in the left ICA-C4-segment occurred leading to a perfusion stop (A*: black colored areas*) and adjacent critical perfusion (A: grey colored areas) in the dorsal left MCA- and nearly all parts of the left fPCA-territory. Thrombotic material was depicted in a major M2-branch of the left MCA (C*, white arrow*) and in a P2-branch (D*, black arrow*) of the fPCA in SWI. Due to the severe perfusion restriction an early ischemic injury of the affected tissue became rapidly evident (B*, bright areas in left hemisphere*). cTE, cerebral endovascular thrombectomy; DWI, diffusion weighted; fPCA, fetal posterior cerebral artery; ICA, internal carotid artery; ivTL, intravenous thrombolysis; MCA, middle cerebral artery; P-MRI, perfusion magnetic resonance imaging; stdTTP, standardized time-to-peak parameter; SWI, susceptibility weighted.

IvTL was started 3.5 hours after onset and the patient was transferred to the intStC. During transport the patient's condition deteriorated leading to a NIHSS of 24 points at admission at the intStC 5.5 hours after onset. Therefore, optimised multiparametric MRI including diffusion weighted (DWI), quantitative P-MRI, susceptibility weighted (SWI) and extended fluid attenuated imaging as well as MR angiography (MRA) was performed (total examination time: 12:53 minutes) [9]. MRA revealed a complete recanalisation of the left ICA and migration of the initial thrombus to the major central M2-branch of the left MCA and a partial filling of a left fPCA became visible (Fig. 1B). SWI suggested migration of thrombus material to the major central M2-branch of the left MCA and to the distal P2-Segment of the left PCA. A severe perfusion drop with rapidly evolving signs of ischemic injury in the left hemisphere was found in stdTTP-maps and DWI (Fig. 2).

**Fig. 3:**
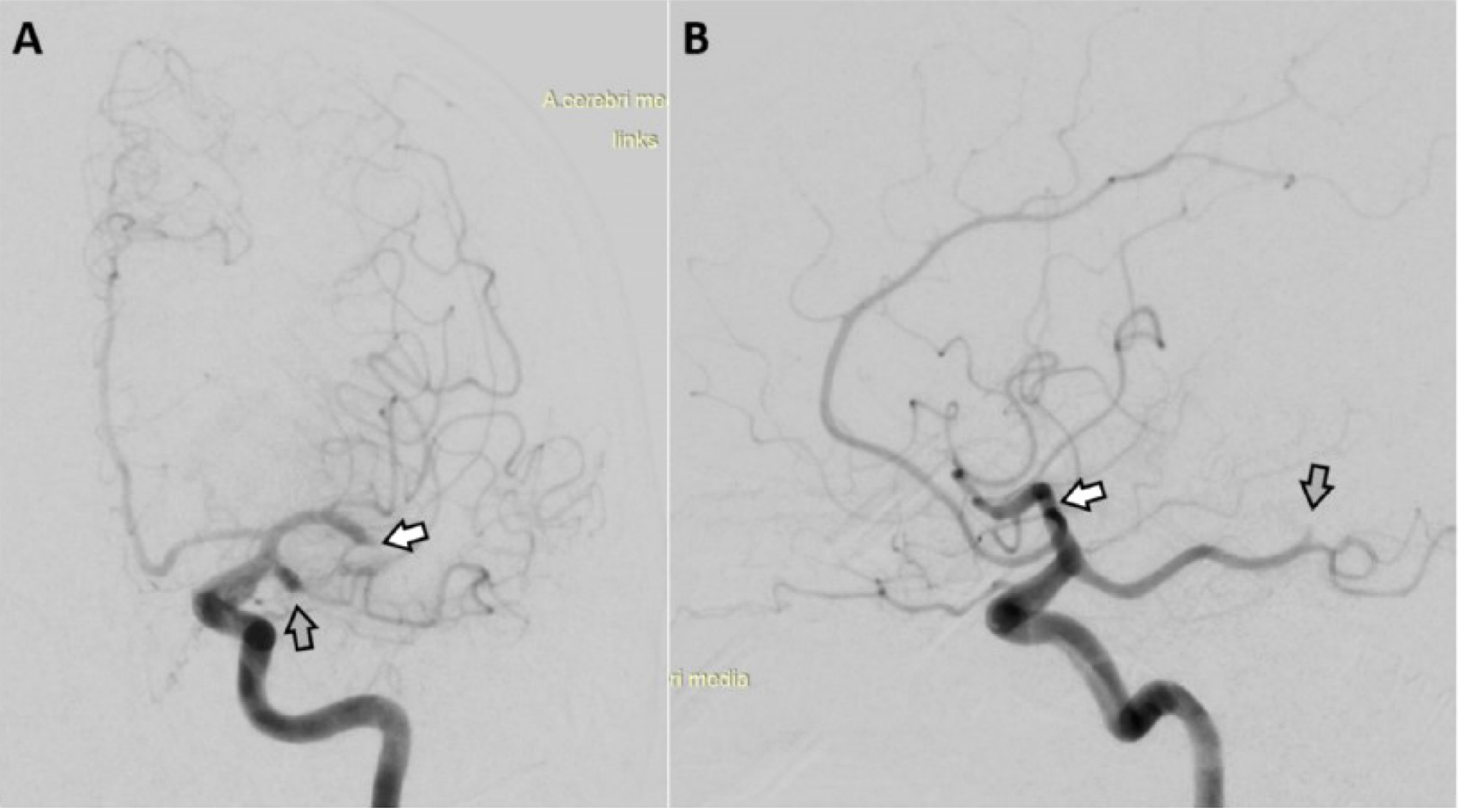
Case 1 - IaDSA performed 6 hours after symptom onset showed a TICI 0-occlusion of the major M2-branch of the left MCA (A, B *white arrow*) and a TICI 0-occlusion of a distal P2-branch arising from a homolateral fPCA (A, B *grey arrow*). fPCA, fetal posterior cerebral artery; iaDSA, intra-arterial digital subtraction angiographic; MCA, middle cerebral artery; TICI, thrombolysis-in-cerebral-infarction.

IaDSA performed 6 hours after onset of symptoms confirmed the findings of multiparametric MRI showing a TICI 0-occlusion of the major M2-branch of the left MCA and a TICI 0-occlusion of a distal P2-branch extending from a homolateral fPCA (Fig. 3). CTE performed in the left MCA using a 4/20 mm Trevo ProVue stent retriever (Stryker Neurovascular, US) allowed extraction of a 5 mm piece of thrombus material. A hostile aortic arch type 3 and massive atherosclerosis of the pelvic vessels prohibited further stable positioning of an appropriate access sheath in the left ICA. Subsequently the patient showed only minor improvement to NIHSS 19 after cTE and complete infarction of the initially critically perfused areas in the left hemisphere was found in follow-up MRI. After 8 weeks of multiprofessional neurorehabilitation the patient showed minor improvements only. At discharge the patient still was severely dependent (mRS 4) and had to be admitted to a nursing care facility.

### Case 2

**Fig. 4:**
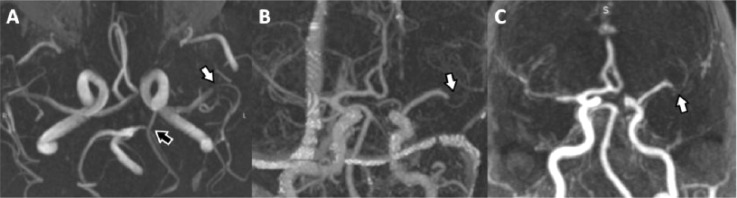
Case 2 - Partial occlusion of the majority of the left MCA-M2-branches was already known from MRA performed several years before the reported acute event (A*, axial view, white arrow*). Acutely performed CTA (B*, white arrow*) and MRA (C*, white arrow*) confirmed this finding. Note that an initially visible fPCA (A*, axial view, open black arrow*) was no longer visible in the acutely performed examinations. CTA, CT-angiography; fPCA, fetal posterior cerebral artery; MCA, middle cerebral artery; MRA, MR angiography.

The 77-year-old male was admitted to the adStU suffering a wake-up stroke, where he was last seen well 8 hours before. On admission he presented with a severe left hemispheric anterior circulation syndrome (NIHSS 19). Initial CT showed demarcated old infarctions in the frontal opercular regions on both sides. CTA depicted an old occlusion of the major M2-branches of the left MCA already known for several years at this time. A previous MRI performed 4 years before had demonstrated a full hemodynamic compensation of this chronic MCA-M2 occlusion and the existence of a left fPCA. In the acute CTA examination this fPCA was not visible and therefore assessed to be completely occluded (Figs. 4A and B).

**Fig. 5:**
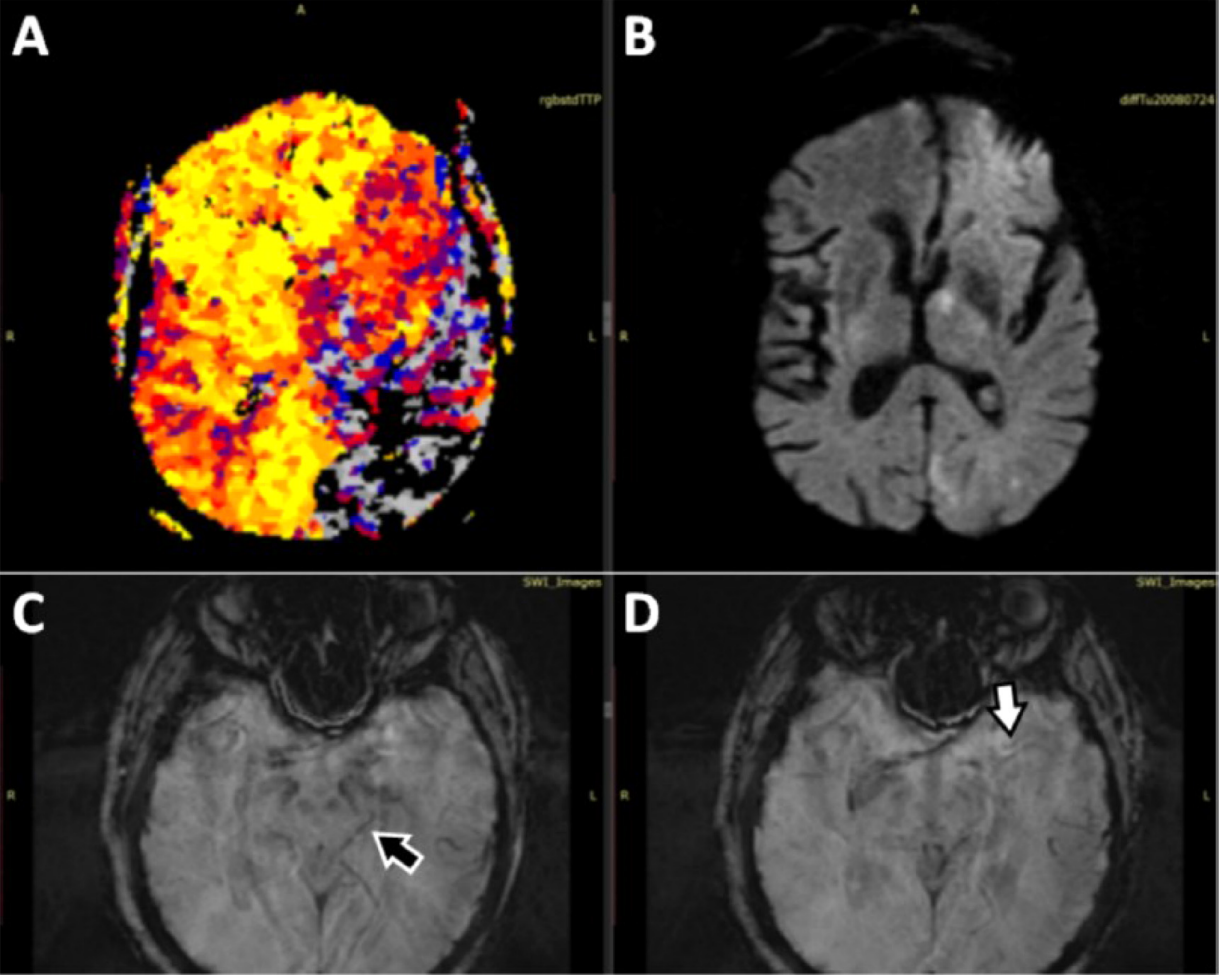
Case 2 - Geometrically matched stdTTP- (A*: regions with a critical residual perfusion appear grey, while black colored regions mark areas with a perfusion stop*) and DWI-maps (B) of case 2 showed a significant mismatch in the left occipital and temporal lobe, rather suggesting acute ischemia in the left PCA-territory. No thrombus material was detectable in SWI, neither in the course of the fPCA (C*: black arrow*) nor the MCA on the left side (D*: white arrow*). DWI, diffusion weighted; fPCA, fetal posterior cerebral artery; MCA, middle cerebral artery; stdTTP, standardized time-to-peak parameter; SWI, susceptibility weighted.

Anticoagulant therapy with apixaban 5mg bid due to atrial fibrillation prohibited bridging ivTL and the patient was urgently transferred to the intStC. There, to prove the state of viability of the ischemic tissue about 3 hours after notice of symptoms, multiparametric MRI comparable to case 1 was performed. Like CTA, MRA depicted also a lack of contrast filling of the major M2-branches on the left side (Fig. 4C). Furthermore, DWI and stdTTP-maps showed a significant mismatch in the left occipital and temporal lobe suggesting an infarction of the left PCA-territory. No thrombus material was detectable in SWI (Fig. 5).

**Fig. 6:**
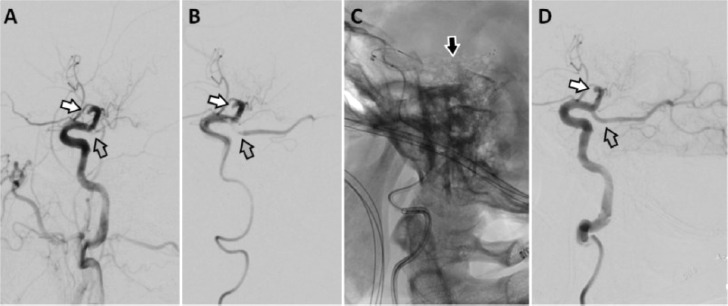
Case 2 - IaDSA in case 2 confirmed the occlusion of nearly all MCA-M2- branches (A,B,D, white arrows). Initially the known fPCA was not visible in iaDSA (A, grey arrow). Manipulation with the micro wire led to a partial recanalisation of the fPCA (B*, grey arrow*) and after cTE using a stent retriever (C*, black arrow*) a full recanalisation of the fPCA was achieved (D*, grey arrow*). cTE, cerebral endovascular thrombectomy; fPCA, fetal posterior cerebral artery; iaDSA, intra-arterial digital subtraction angiographic; MCA, middle cerebral artery.

**Fig. 7:**
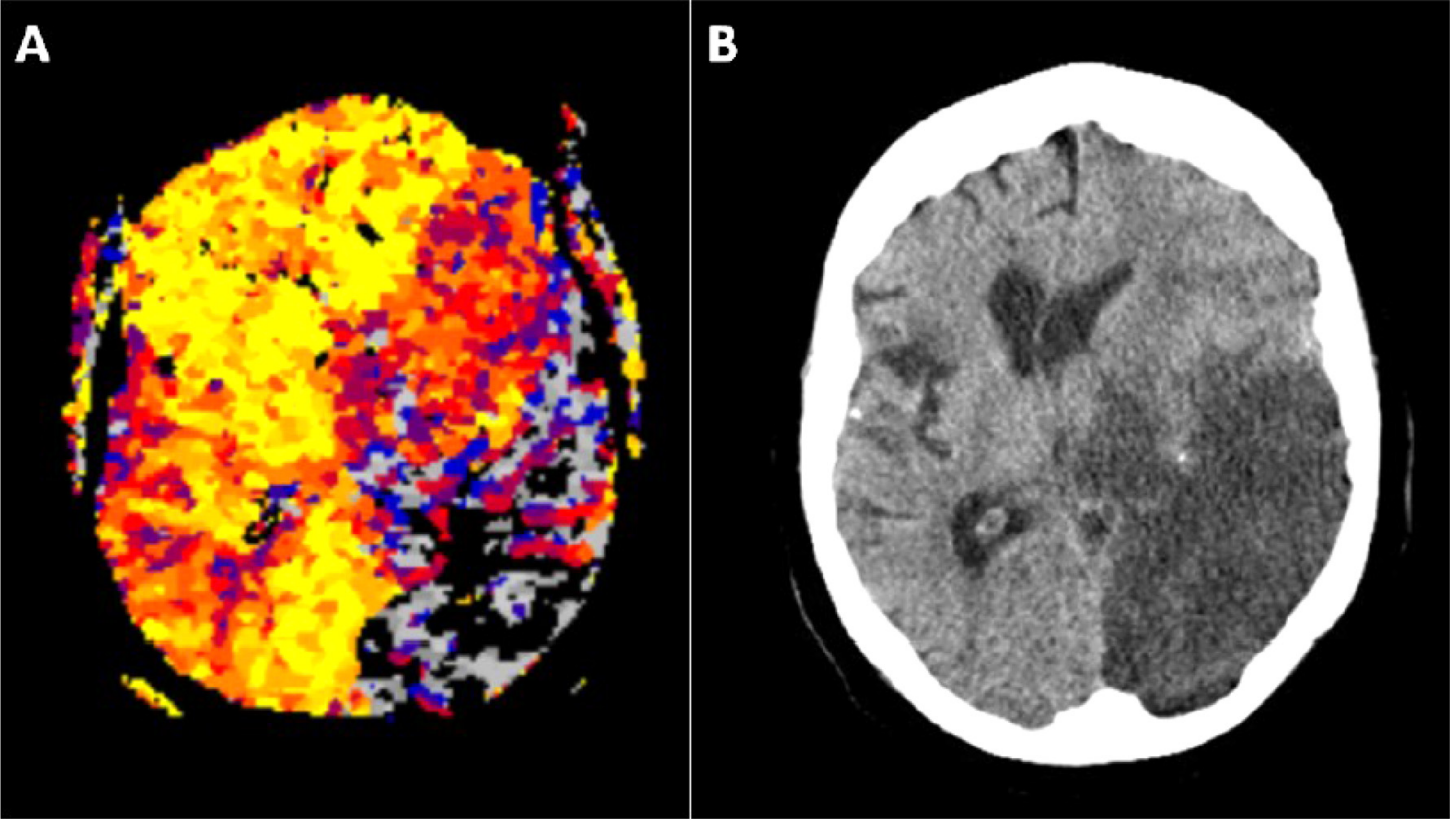
Case 2 - Due to recurrent reocclusions of the left fPCA and resulting prolonged intervention time in case 2 the critically perfused areas in the PCA-territory depicted in initial P-MRI before the intervention (A*: grey and black colored brain areas*) progressed to complete infarctions with severe brain swelling shown by CT several days after the acute event (B). fPCA, fetal posterior cerebral artery; P-MRI, perfusion magnetic resonance imaging.

IaDSA confirmed the occlusion of the majority of the left MCA-M2 branches. Additionally, thrombotic material adherent to vessel wall at the transition between the C2- and C1-segment of the left ICA occluded the left fPCA. After probing at this location with a microwire (Synchro 014, Stryker Neurovascular, US) successful cTE of the fPCA using a stent retriever (4/20 mm Trevo ProVue, Stryker Neurovascular, US) could be performed with a TICI 3 result after 5h 20 min after notice of symptoms (Fig. 6). CTE of the left ACM-M2 occlusions was not tried, as these were already known for several years. In the course of the procedure recurrent embolisations into the territory of the left fPCA occurred requiring retreatment with cTE, which prolonged the intervention and degraded the final result to TICI 2b at 6h 55 min after notice of symptoms. The further course of disease was fatal due to a complete infarction in the left fPCA-territory with massive brain swelling and intractable high intracranial pressure (Fig. 7).

## Discussion

A fPCA is found with higher prevalence in stroke patients than in control subjects (19.35% vs 9.2% min) and could, therefore, be considered an anatomical risk factor for ischemic stroke [12]. Despite this, quite few is known about diversion of cerebral blood flow in case of an occluded fPCA requiring recanalisation. Hemodynamically, the fPCA-territory stays part of the anterior circulation supplied by a strong calibrated persisting caudal portion of the embryonic ICA, which normally regresses to the posterior communicating artery [13]. The PCA-P2 segment sustains from the persistent embryonic ICA, while the PCA-P1-segment is missing or hypoplastic. Since this widely decouples flow between the anterior and posterior circulation [7], the flow in a fPCA is directed from the ICA to the PCA-territory. Nevertheless, the major ICA flow component still points toward the homolateral ACA- and MCAterritories, since the active vascular cross section of the distal ICA is usually larger than that of the fPCA. Therefore, in a typical fPCA variant, thromboembolism exclusively to the fPCA territory is extremely rare unless other flow modifications occur [1,14].

In our cases thromboembolism of the fPCA was observed in combination with occlusion of the majority of the homolateral MCA-M2 branches suggesting a flow diversion from the cranial to the caudal part of the ICA. While in case 1 an initial embolism to the homolateral MCA remains speculative, the redirection of the embolic trajectory from the cranial ICA portion to the fPCA is evident in case 2, where homolateral MCA-M2 occlusions had been previously documented. Thus, it is conceivable that in case 1 embolism into the M2-branches also occurred prior to the fPCA-occlusion. However, multi-modal MRI performed immediately before thrombectomy revealed large mostly critically perfused fPCA-territories with rapidly progressing extensive infarct cores (Figs. 2 and 5) in both cases. The big size of the critically perfused fPCA-territories, finally triggering unfavourable outcomes, is well explained by the blockage of leptomeningeal MCA-collaterals and the general lack of collateral blood flow from the posterior circulation in the fPCA-variant [7,15].

Modelling of cerebral flow dynamics additionally supports this assumption, where a complete collapse of blood flow in the fPCA-territory was found when, comparable to the initial situation of case 1, an occlusion of the homolateral ICA was simulated [16].

Though in both cases recanalisation of the fPCA was achieved quickly by ivTL and cTE respectively, a severe lack of collateral blood flow together with the postulated unfavourable shift of the embolic trajectory from the cranial ICA to the fPCA seems to potentially limit the success of treatment in case of an occluded fPCA. Concordantly, reported success rates for endovascular and intravenous treatment of fPCA occlusions remain variable, ranging from successful stable recanalisation to fatal courses of ischemia due to malignant brain oedema [1,14,17]. Unlike than in occlusion of large cerebral vessels, namely, the intracranial ICA, the proximal MCA or the basilar artery, endovascular treatment of an occluded fPCA is currently not explicitly recommended [18]. However, especially in case of a proven large critically perfused fPCA-territory, as shown here, endovascular treatment is feasible and should be considered, because outcome may be fatal without a stable successful recanalisation. Though meanwhile endovascular treatment of various types of fPCA occlusions was reported feasible in literature, reliable data from prospective trials are still pending [1,14,17]. Furthermore, the choice of the most appropriate technique for cTE (direct thrombus aspiration, use of a stent retriever etc.) also remains of concern, since the actual thromboembolic trajectory has to be considered individually in each patient [1].

Nevertheless, we believe that our presented case histories provide additional important learning points to published knowledge so far:

1) Collateral supply to an occluded fPCA territory was shown to rapidly become insufficient, particularly, in conjunction with a partial or complete collapse of blood flow in the residual anterior circulation. Therefore, the timely and complete stable recanalization of the fPCA seems of utmost importance.
2) Flow restrictions in the distal anterior circulation may shift the embolic trajectory from the cranial ICA-portion to the fPCA, thereby facilitating recurrent embolism to the fPCA. Thus, a stable recanalisation of the fPCA seems to require regular flow dynamics also in the ACA- and MCA-territories.
3) Endovascular treatment of an occlusion of an fPCA is feasible, but faces most often a complex hemodynamic situation.

In conclusion neurologists and neuroradiologists should consider the possibility of ischemic stroke in an fPCA-variant, especially in case of a severe affection of an unexpectedly large PCA-territory. Since collateral supply may be very low, a fast and experienced approach, including endovascular treatment, to reopen the occluded vessel seems necessary.

## Ethical considerations

This study was conducted according to the Helsinki Declaration.

## Consent for publication

All authors declared their consent to publication of this work.

## Availability of data and materials

Not applicable.

## Patient consent

Written informed consent was obtained for the publication of this case report.
